# Occurrence of Multidrug-Resistant Strains of *Acinetobacter* spp.: An Emerging Threat for Nosocomial-Borne Infection in Najran Region, KSA

**DOI:** 10.3390/tropicalmed8020108

**Published:** 2023-02-09

**Authors:** Abdullah I. Aedh, Ali Dhafer Al-Swedan, Asiri Ahmed Mohammed, Batool Mubarak Alwadai, Ahlam Yahya Alyami, Esraa Amer Alsaaed, Nouf Mubarak Almurdhimah, Mohamed Soliman Zaki, Alyaa E. Othman, Abdulkarim Hasan

**Affiliations:** 1 Consultant of Internal Medicine and Critical Care, Department of Internal Medicine, Najran University Hospital, Najran University, Najran 55461, Saudi Arabia; 2 Infectious Diseases and Internal Medicine, King Khalid Hospital, Najran University, Najran 55461, Saudi Arabia; 3 Senior Medical Residents, King Khalid Hospital, Najran University, Najran 55461, Saudi Arabia; 4 Department of Chest Diseases, Faculty of Medicine, Al-Azhar University, Cairo 11884, Egypt; 5 Department of Infectious Diseases, Faculty of Medicine, Suez Canal University, Ismailia 41522, Egypt; 6 Department of Pathology, Faculty of Medicine, Al-Azhar University, Cairo 11884, Egypt; 7 Department of Laboratory Medicine, Prince Mishari bin Saud Hospital, Saudi Ministry of Health, Baljurashi 2288, Saudi Arabia

**Keywords:** *Acinetobacter*, antimicrobial-resistant, epidemic infection outbreaks, nosocomial-borne infection

## Abstract

Multidrug-resistant strains are frequent causes of nosocomial infections. The majority of nosocomial infections, particularly in critical care units (ICU), have been linked to *A. baumannii*, which has major clinical significance. The current paper attempts to identify the potential risk and prognosis factors for acquiring an infection due to *A. baumannii* compared to that of other nosocomial bacteria. In our study, we employed antibiotics generally prescribed for the initial course of treatment such as colistin, meropenem, amikacin, trimethoprime-sulfamethoxazole, levofloxacin, gentamicin, ciprofloxacin, and piperacillin-tazobactam. We found that the isolated *A. baumannii* were resistant at a high rate to meropenem, piperacillin–tazobactam, amikacin, levofloxacin, and ciprofloxacin, while they were partially susceptible to trimethoprim-sulfamethoxazole. Our study revealed that *A. baumannii* was most susceptible to gentamicin and colistin at 85.8% and 92.9%, respectively, whereas the combination of colistin and trimethoprim/sulfamethoxazole was 100% active. The patients were the primary source of infection with *A. baumannii*, followed by inanimate objects present in the ICU and hospital premises, and then the hospital staff who were taking care of the ICU patients. Gentamicin and colistin were the most sensitive antibiotics; of the 13 tested in total, the rate of drug resistance was above 50%. The very high rate of antibiotic resistance is alarming.

## 1. Introduction

Nosocomial infections (NIs) are communal, and they are associated with numerous bacteria that are ubiquitous in hospital zones. These hospital-borne infections are associated with high rates of mortality and morbidity [1]. It was more common for NIs to be bacterial in origin, and the most prevalent sources were the urinary tract, circulatory system, abdomen, and brain [2]. A higher rate of infection was shown to be associated with the prolonged use of parenteral feeding in addition to the time spent in the ICU [3]. To be properly treated, early detection and treatment for such infectious diseases are essential to ensure the best outcome [4]. Both Gram-negative and Gram-positive MDR bacilli are frequently seen in NIs. In selecting a preventive strategy, the prevalent bacterial isolates found in specific hospitals must be considered [5].

This kind of hospital-borne infection is more common in the USA and Middle Eastern nations due to the emergence of new antibiotic-resistant species in the hospital itself [6]. There should be a routine examination of infections and their resistance. Nosocomial surgical site infections (NSSI), nosocomial urinary tract infections (UTIs), nosocomial bloodstream infections (NBSIs), and nosocomial pneumonia (NPNEU) accounted for 80% of all nosocomial infections (NI) [7]. The rate of NI incidence has changed over time and across geographical regions; for every 100 patients, the World Health Organization (WHO) reported that seven patients from industrialized nations and ten from underdeveloped areas were suspected of having a nosocomial illness [8].

Antibiotic-resistant strains are the most frequent causes of infection. The most frequently resistant strains are coagulase-negative *Staphylococci*, methicillin-resistant *Staphylococcus aureus* (MRSA), *Enterococci*, etc [8,9,10]. According to a recent report, the presence and dissemination of infectious material, which includes endogenous organisms as well as exogenous or cross-environmental infections, is a significant source of these diseases [11]. Vancomycin-resistant *Enterococcus* (VRE) was identified as the most hazardous pathogen in the hospital setting [12]. According to recent studies from Middle Eastern countries, more resistant strains of *Acinetobacter* spp. were reported as a prevalent contributor to resistance-acquired nosocomial infections [7]. In nature, water, and soil, Gram-negative coccobacilli, known as the *Acinetobacter* species, are common [13,14]. *Acinetobacter* includes about 20 different species. However, only a handful, such as *Acinetobacter baumannii* (*A. baumannii*), *A. calcoaceticus*, and *A. lwoffii*, are considerable contributors to nosocomial infections [15]. *A. baumannii* is a Gram-negative bacillus that is a well-known aerobic, pleomorphic, and non-motile organism. It is an opportunistic pathogen, commonly associated with aquatic environments, that has a high incidence among immunocompromised individuals, particularly patients who have experienced a prolonged (more than 90 days) hospital stay [16].

*A. baumannii* has become a predominant nosocomial pathogen, particularly in critical care units (ICU) [17,18,19,20]. Numerous infections, including bacteremia, meningitis, infections of the urinary tract, bloodstream, or surgical wounds, and ventilator-associated pneumonia, have also been reported to be caused by *A. baumannii* [21,22].

*Acinetobacter* spp. is a significant pathogen in epidemic outbreaks as well as in endemic colonization. Due to the numerous characteristics causing *Acinetobacter* opportunistic infections, these pathogens have been reported to cause a variety of diseases. *A. baumannii* infections have been studied extensively; however, only a small number of reliable reports globally have attempted to assess their clinical significance [21]. The lower the distribution of the locations of *Acinetobacter* infections, the more likely to affect the respiratory system and urinary tract. The current paper attempts to identify the potential risk and prognosis factors of *A. baumannii* compared to that of other nosocomial bacteria. Patients who are in susceptible groups, such as those with compromised immune defenses, are more at risk. Because of its propensity for long-term survival, *A. baumannii* can spread quickly in medical facilities. These characteristics may determine its tendency to cause long-lasting epidemics. Direct contact with infected people or indirect interaction via contaminated environments is the main way that *A. baumannii* spreads. Thus, it was generally assumed that *A. baumannii* transmission occurs due to interactions between patients, healthcare providers, and contaminated fomites in the hospital’s environment; *A. baumannii* was not considered an airborne droplet bacillus despite causing outbreaks of community-acquired pneumonia in some countries [23,24].

However, the airborne route also contributes significantly to *A. baumannii* transmission in hospitals. Despite the fact that airborne transmission is thought to be a method of acquiring *A. baumannii* infections, there are not many studies in this area [23]. Therefore, it is necessary to put control measures in place to limit the spread of this pathogen within the hospital setting. It is also advised that healthcare facilities put in place suitable safety measures to prevent the spread of these and other dangerous microorganisms. This current clinical report investigates the prevalence of *Acinetobacter* spp. in King Khalid hospital, Najran, as a surveillance study conducted to monitor the resistance pattern acquired in the hospital environment.

## 2. Materials and Method

### 2.1. Study Setting

The current study was investigated as a cohort observational study obtained from the information gathered from the data sheets of the patients in the intensive care unit at King Khalid Hospital, Najran, a tertiary care facility in the southern part of Saudi Arabia, between January 2022 and June 2022.

Patients who were admitted to the ICU for more than 48 h and had a hospital-acquired infection made up the study group. The demographic, microbiological, antimicrobial treatment and patient outcome data were gathered. In this investigation, 532 patients with bacteremia, pneumonia, and other infections were categorized as nosocomial infections (those developing within 48 h of hospital admission) or illnesses acquired through healthcare.

### 2.2. Bacterial Isolates and Antibiotic Susceptibility

Clinical samples from various illnesses in the ICU (*n* = 79) were prepared for culture using traditional techniques (cultures on routine media such as blood agar or MacConkey agar and selective media with specific biochemical tests when necessary) where all the clinical isolates have proceeded for culture on blood and MacConkey agars and then Gram staining was performed to identify bacterial morphology and Gram reaction. The studies were cultured several times to ensure that all isolates were pure. Several biochemical tests were performed to confirm that all isolates belonged to *A. baumannii*, including oxidase, catalase, and indole tests. Standard phenotypic assays were used for the initial identification [25].

Vitek 2 system (BioMerieux, Marcy-l’Étoile, France) was used in the microbiology laboratory to confirm the identification of the isolates, following the manufacturer’s instructions. All isolates were investigated for antibiotic susceptibility, using this automated Vitek 2 Compact system. The included samples were cultured on blood agars, and then the suspension was made for every single isolate. A liquid suspension of the studied isolates was loaded on the Vitek system, and left overnight to obtain the result. The next day, results illustrated the samples’ identification and antibiotic susceptibility.

VITEK 2 system was approved for authenticating the names of *Acinetobacter* spp. as described by the manufacturer (BioMerieux). The Vitek2 card contains 64 wells holding different fluorescent biochemical assays. Twenty out of the sixty-four wells were carbohydrate assimilation; four are phosphatase, nitrate, urea, and actidione tests. The machine controlled this card automatically, including filling, sealing, and finally transferring such cards into the linked incubator at a temperature of 35 °C. Each output report is usually decoded according to a specific algorithmic system. The acquired results were recognized and recorded. Most known *Acinetobacter* spp. have clear-cut profiles, and the system led to the correction of the unknown organism.

The susceptibility of the most commonly used antibiotics (*n* = 13) for the prevalent ICU infections has been recorded.

### 2.3. Ethical Approval

The Najran University, Faculty Of Medicine, Ethics Internal Review Board Committee provided ethical approval (No. 10/1/22/NU/DS Date: 3 January 2022).

### 2.4. Statistical Analysis

Statistical variables, including the biodemographic information, the source of the clinical specimens, and the type of organisms were subjected to descriptive analysis (cross tab, frequency, and proportion) and were used to compare the trend of the antimicrobial resistance rate, the prevalence of the MDR *A. baumannii*, and its resistance rates, using Microsoft Excel 2019. The statistical analysis was performed using SPSS version 26 software (SPSS Inc, Chicago, IL, USA) and *p* values < 0.05 were considered statistically significant.

## 3. Results

The global rise of antimicrobial-resistant strains of hospital-borne pathogens is a threat since those strains are difficult to eliminate with common prophylaxes. In such conditions, it is essential to stringently monitor the emergence of new antibiotic-resistant strains due to their lethality. Many pathogens have been reported to cause a strong impact on clinical settings; however, the recently reported resistant strains of *A. baumannii* have attracted particular attention. Table 1 depicts the current study, where, out of 532 samples, 79 were collected from the ICU; some strains were found to have the infection *Acinetobacter* spp. It is an omnipresent coccobacillus showing Gram-negative characteristics under light microscopy staining. Among the 79 samples, we isolated 28 samples of *A. baumannii* and the rest revealed non-*Acinetobacter* spp. where a significant difference between the two groups was seen in the (wound) infection site; however, the rest of the sites showed no statistical significance. Additionally, giving a history of respiratory disorders and a history of diabetes mellitus disease showed a significant difference between *Acinetobacter* and non-*Acinetobacter* spp groups. Specifically, non-Acinetobacter spp were isolated at a higher rate from wounds and were associated more commonly with a medical history of diabetes mellitus followed by a history of respiratory disorders.

Among 79 consecutive hospitalized ICU patients during the 6-month study period, 28 (35.4%) developed an *A. baumannii* infection, and 51 (64.6%) developed non-*Acinetobacter* infections in this study. The age group of the 79 patients involved in this study ranged from 17 to 48. The mean age of the total number of males was 48.4 ± 4.4 and females was 42.8 ± 4.6 with no significant difference. Among the 79 (100%) clinical isolates, the percentage isolated from males was 57/79 (72.1%) and from females was 22 (27.8%). *A. baumannii* was isolated from 21 male samples (26.5%) and 7 female samples (8.8%), whereas the non-*A. baumannii* group comprised 36 (45.5%) males and 15 (18.9%) females. From the ICU, the clinical isolates included: 36 (45.7%) from a wound, 8 (10.1%) from the blood, 11 (13.9%) from the respiratory tract, 10 (12.6%) from the central venous catheter, 2 (2.5%) from the surgical area, and 12 (15.1%) from the urinary tract. For the *A. baumannii,* there were 10 (12.6%) from a wound, 3 (3.7%) from the bloodstream, 4 (5%) from the respiratory tract, 5 (6.3%) from the central venous catheter, 1 (1.26%) from the surgical site, and **5** (6.3%) from the urinary tract.

The total number of clinical isolates was categorized based on the patient’s medical history including 13 (16.4%) patients without any medical issues, 4 (5%) post-cardiac arrest, 13 (16.4%) with a respiratory infection, 38 (48.1%) with diabetes mellitus, 7 (8.8%) with corticosteroid use, 3 (3.7%) with a bomb blast injury, and 1 (1.26%) with osteomyelitis; *A. baumannii* were isolated from 4 (5%) patients without any medical history, 4 (5%) with cardiovascular issues, 2 (2.5%) with a respiratory infection, 11 (13.9%) with diabetes mellitus, 3 (3.7%) with corticosteroid use, 3 (3.7%) with a bomb blast injury, and 1 (1.26%) with osteomyelitis.

We present the significance of infections caused by *Acinetobacter baumannii* and their antibiotic susceptibility pattern in our study conducted over a six-month period (January 2022 and June 2022) at a tertiary-care super-specialty hospital. The resistance rates to antimicrobial agents are presented in Table 2. Colistin was the most active antimicrobial agent (92.9% susceptibility). Multi-resistant *A. baumannii* were identified in 23/28 patients who comprised the multi-resistant group; the remaining five constituted the non-multi-resistant group.

In our study, we employed the antibiotics generally prescribed for the initial course of treatment, such as colistin, meropenem, amikacin, trimethoprim–sulfamethoxazole, levofloxacin, gentamicin, ciprofloxacin, piperacillin–tazobactam, and other antibiotics. The twenty-eight clinically isolated *A. baumannii* are listed in Table 2. They were, to a lesser extent, resistant to colistin and gentamicin. We found that the isolated *A. baumannii* demonstrated high rates of resistance to meropenem, piperacillin–tazobactam, amikacin, levofloxacin, and ciprofloxacin, while trimethoprim–sulfamethoxazole was active against 54% of the isolates. One isolate and two others were resistant to all the tested antibiotics except amikacin and piperacillin-tazobactam and tobramycin, respectively. However, the isolated strains in our study revealed gentamicin and colistin were the most active antibiotics at 85.8% and 92.9%, respectively (Table 2).

**Figure 1:**
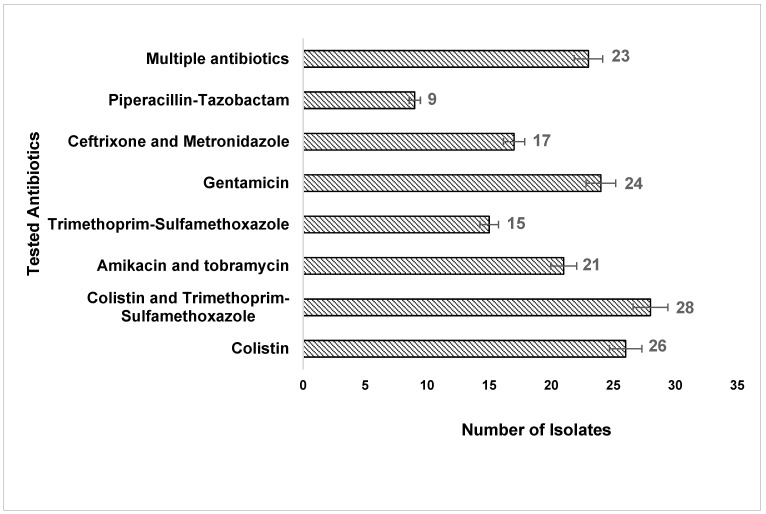
The number of *A. baumannii* isolates susceptible to the tested antibiotics.

Among the clinical isolates, 9 (32%) were not susceptible to piperacillin–tazobactam, whereas, for the highest sensitivity, 23 (82.1%) were susceptible to multiple antibiotics, 24 (85.7%) to gentamicin, 26 (92.8%) to colistin, and 28 (100%) to colistin and trimethoprim/sulfamethoxazole (Figure 1). In this context, our current study also observed an isolated resistant subset of *A. baumannii* from various clinical specimens of the same patient. However, in many of the patients, we observed the samples showing two different antibiograms, and further analysis indicated that the isolated organism is generally commensal; in our study, we found that it is clinically correlated as a pathogen to a further extent and might be dangerous in future as the current isolate exhibits a few resistant characteristics.

## 4. Discussion

*A. baumannii* is a Gram-negative omnipresent coccobacillus opportunistic pathogen as the *Acinetobacter* spp. is typically an opportunistic pathogen. In recent years, a variety of nosocomial infection outbreaks in hospitalized patients, including septicemia, pneumonia, wound sepsis, endocarditis, meningitis, and urinary tract infection (UTI), have been recorded. Community-acquired infections are opportunistic in hospitalized patients and can result in suppurative infections in every organ system [26]. Due to their widespread nature, they colonize both healthy and injured tissue, and understanding the implications of their presence in clinical specimens is important [27]. There were Gram-negative bacilli found on the skin of up to 25% of healthy ambulatory hospital staff who demonstrated cutaneous colonization [28]. We presented the significance of the infections caused by *A. baumannii* and their antibiotic susceptibility pattern in our study conducted over a six-month period (January through June 2022) at a tertiary-care super-specialty hospital. A small set of resistant subpopulations of organisms that have already colonized people benefit from exposure to specific antibiotics, which makes it easier for them to transition into pathogens when the conditions are right. In this context, our current study also observed an isolated resistant subset of *A. baumannii* from various clinical specimens from the same patient. However, in many of the patients, we observed samples with two different antibiograms, and further analysis indicated that the isolated organism was generally commensal; in our study, we found that it was clinically correlated as a pathogen, and it might be dangerous in future, as the current isolate exhibited a few resistant characteristics. These results are consistent with other peer studies in the Middle East revealing a lower resistance rate for colistin and for multiple antibiotics. A study in Qatar stated that the maximum resistance of *Acinetobacter* spp. was seen to cefotaxime, and minimum resistance was seen to colistin [29]. A recent study from Oman on *A. baumannii* strains showed high resistance to most of the tested antibiotics; the highest was against ceftriaxone (83% of cases) and ceftazidime (75%), whereas the lowest resistance was against tigecycline (8%) and colistin (1%). Among the isolates, 67% were MDR strains [30]. In Saudia Arabia, a recently published study indicated resistance in more than 80% of *A. baumannii* isolates to the carbapenems, whereas the isolates were found almost totally sensitive to colistin and only 5% of isolates were resistant to the tigecycline [31]. In our study, tigecycline was not tested. In a study from Jordan, tigecycline and colistin also had the lowest resistance rates of *A. baumannii*; however, 76.8% of isolates were MDR and 99% were carbapenem-resistant [32]. In the Asian country Malaysia, colistin was the most commonly used antibiotic for carbapenem-resistant *A. baumannii* isolates [33]. Alarming results, which are different from ours, came from Egypt where the colistin resistance was up to 20% [34].

According to the Infectious Diseases Society of America, in the USA, *A. baumannii* is a “red alert” pathogen that dramatically endangers the efficacy of the present arsenal of antibiotics [35]. As a result of resistance, only a few antimicrobials can be used consistently for successful MDR *Acinetobacter* infection treatment.

When treating nosocomial *Acinetobacter* infections, few antimicrobials are active; hence, the review of outdated medications and the development of new ones have become a concern [36].

We analyzed the sex specification of the incidence of infection, and we found that more than 70% were male patients admitted to the ICU. The incidence with respect to female patients was lower (Table 1). We still do not understand the pattern of *Acinetobacter* infection, however, as the number of males included in this study was higher than the number of females. Moreover, we observed the incidence of this infection was not associated with co-morbidities. This suggests that this incidence was purely nosocomial-borne infections, not specifically associated with any chronic co-morbidities.

On cross-examining the clinical case sheets of the patients, we found that in all the subjects, peripheral catheterization was a common pattern observed in the case of *A. baumannii*, irrespective of sex. Therefore, this could be either due to the hospital’s hygiene or the lack of expertise in the ward attending nurse who may carry this kind of infection. We also found that the infections were caused by a minimum stay in the hospital ICU. The length of the stay of the subjects was not more than 3 months. This was also notable with respect to the incidence of *A. baumannii* infection.

However, there have been several recent advances toward improving the safety and efficacy of the current therapeutic options for *A. baumannii* infection; unfortunately, there remains a paucity of recent high-quality clinical data to inform the optimal treatment of MDR *A. baumannii* infections.

In fact, the recently completed studies failed to identify even a combination regimen that is supposed to be consistently superior to monotherapy, depending on the benefits demonstrated in vitro [37].

β-Lactams are considered drugs of choice against susceptible *A. baumannii* infections. However, just 26% of MDR *A. baumannii* infections in the USA remain susceptible to one or more first-line agents, including carbapenems or sulbactam [38,39]. Polymyxins also became a backbone in the treatment of MDR *A. baumannii* infections over the last few decades, and among available studies, the mortality rates reported from severe MDR *A. baumannii* infections treated with those polymyxins cluster between 30–60%, in part reflecting the frequent limitations of many clinical trials on polymyxins, such as the lack of standardized testing and dosing of polymyxin susceptibility [40].

Tetracyclines look like polymyxins, and there has been a renewed interest in using tetracyclines against *A. baumannii* infections due to the potent activity of tetracyclines against *A. baumannii* infections, especially MDR strains. Additionally, aminoglycosides are still considered one of the most active antimicrobials in vitro against *A. baumannii* infections, with approximately 80% of the total isolates retaining susceptibility against *A. baumannii* [37].

The prevalence and rate of increased MDR-acquired infections, particularly colistin resistance in Eastern Mediterranean and South-East Asian countries, are higher than in other regions of the world [41,42]. Accordingly, there are still no standard therapeutic recommendations for the management and control of MDR, and colistin-resistant Acinetobacter infections.

Our study revealed that colistin (92%) and gentamicin (85%) were the most active antibiotics, whereas, in earlier studies, the results of the spectrum rates of colistin (60%) and trimethoprim and sulfamethoxazole (46%) did not correlate with our studies [43,44]. Among the clinical isolates, *A. baumannii* was extremely resistant to two carbapenem drugs, meropenem (57%) and imipenem (53%), and these spectrum rates were not correlated with previous studies [45], indicating that the spectrum of the antimicrobial resistance profile may vary from place to place and nosocomial-associated pathogens. This study revealed that these clinical isolates were more risky than the previously identified *A. baumannii*, which showed changes in resistance [39]. The overall prevalence of MDR among the clinical isolates was 82.1%, which was comparable to Bosnia and Herzegovina (78.4%) [46].

Shamsizadeh et al. [47] reported in their clinical study that the hospital premises were the major reason for the hospital-borne outbreaks of *Acinetobacter* infections, where this strain of *A. baumannii* was able to live for a long duration in patient beds in the ICU. According to the previous study’s findings, multidrug-resistant *A. baumannii* was found in the air, water, and surface areas of hospitals. Therefore, it is essential to identify *A. baumannii* infections early and put appropriate control measures in place to stop their spread throughout hospital settings, particularly in ICUs [35].

The limitations of the study findings included: (1) factors such as the number of ICU beds, hospital beds, and the types of patients admitted to the hospital (veteran and non-veteran patients) that may influence the rate of MDR *Acinetobacter* infection; (2) the infection rates may vary between different hospital settings in the same country; and (3) the emergence of antibiotic resistance with *A. baumannii* appears to be worsening, leaving colistin or a combination with colistin as the last effective medication.

## 5. Conclusions

Among the 79 ICU clinical isolates of this study in the Southern area of Saudi Arabia, the overall incidence of *Acinetobacter* infection was 35.4%, and all of these were *A. baumannii*. Our study revealed gentamicin and colistin were the most sensitive antibiotics; the rate of resistance to all other antibiotics tested was above 50% with the exception of trimethoprim-sulfamethoxazole, which was active against 50% of the isolates. The very high rate of antibiotic resistance is alarming; therefore, the utmost care must be taken, failing to do so would mean that such a pathogen would become a serious threat to patients with compromised immunity and long-term stays in ICUs. Aggressive infection control strategies in ICUs with strict follow-up and further studies, including genetic testing, are needed.

## Figures and Tables

**Table 1:** Demographics and clinical data of the total study group, including the *Acinetobacter* and non-*Acinetobacter* species isolated from the ICU.

Variable	Total Group (*n* = 79)	*A. baumannii* (*n* = 28)	Non-*Acinetobacter* spp. (*n* = 51)
Total number of isolated microorganisms from ICU	79 (100)	28 (35.4%)	51 (64.6%)
Age (years)			
Male Female	48.4 ± 4.4 42.8 ± 4.6	46.8 ± 4.4 40.1 ± 4.2	48.6 ± 3.5 43.5 ± 4.7
Sex			
Male Female	57 (72.1%) 22 (27.9%)	21 (26.6%) 7 (8.9%)	36 (45.5%) * 15 (19%)
Site of the infection			
Wound Blood Respiratory tract Central venous catheter Surgical area Urinary tract	36 (45.5%) 8 (10.1%) 11 (13.9%) 10 (12.6%) 2 (2.5%) 12 (15.1%)	10 (12.6%) 3 (3.7%) 4 (5%) 5 (6.3%) 1 (1.26%) 5 (6.3%)	26 (32.9%) * 5 (6.3%) 7 (8.8%) 5 (6.3%) 1 (1.26%) 7 (8.8%)
Medical history			
No medical history Post cardiac arrest Respiratory Diabetes mellitus Corticosteroids user Bomb blast injury Osteomyelitis	13 (16.4%) 4 (5%) 13 (16.4%) 38 (48.1%) 7 (8.8%) 3 (3.7%) 1 (1.26%)	4 (5%) 4 (5%) 2 (2.5%) 11 (13.9%) 3 (3.7%) 3 (3.7%) 1 (1.26%)	9 (11.3%) * 0 11 (13.9%) * 27 (34.1%) * 4 (5%) 0 * 0

* Represents the statistically significant variables (*p* values were < 0.05) by 2-tailed.

**Table 2:** The in vitro susceptibility of *A. baumannii* to common antimicrobial agents.

Antimicrobial Agent	*A. baumannii* (*n* = 28)	Percentage of Resistance
Ceftazidime	25	89.2%
Cefotaxime	22	78.5%
Cefepime	20	71.4%
Piperacillin/tazobactam	19	67.8%
Imipenem	15	53.5%
Meropenem	16	57.1%
Ciprofloxacin	22	78.5%
Levofloxacin	21	75%
Amikacin	19	67.8%
Tobramycin	16	57.1%
Gentamicin	4	14.2%
Trimethoprim-sulfamethoxazole	13	46.4%
Colistin	2	7.1%
